# Teachers’ Professional Identity and Burnout among Chinese Female School Teachers: Mediating Roles of Work Engagement and Psychological Capital

**DOI:** 10.3390/ijerph192013477

**Published:** 2022-10-18

**Authors:** Changkang Sun, Xuechao Feng, Binghai Sun, Weijian Li, Chenyin Zhong

**Affiliations:** 1 College of Teacher Education, Zhejiang Normal University, Jinhua 321004, China; 2 College of Education and Human Development, Zhejiang Normal University, Jinhua 321004, China; 3 Research Center of Tin Ka Ping Moral Education, Zhejiang Normal University, Jinhua 321004, China; 4 Key Laboratory of Intelligent Education Technology and Application of Zhejiang Province, Zhejiang Normal University, Jinhua 321004, China; 5 Yuxiu Secondary School, Changyi 261300, China

**Keywords:** teacher professional identity, work engagement, teacher psychological capital, burnout, female teachers

## Abstract

Burnout is a serious phenomenon among female kindergarten, primary, and secondary school teachers in China. Previous research has shown that professional identity negatively predicts burnout. However, little is known about the mediating mechanisms underlying this relationship. This study examined the relationship between professional identity and burnout and the mediating roles of work engagement and psychology using a sample of Chinese teachers. A total of 2220 female teachers participated (kindergarten: 16.9%; primary school: 56.7%; secondary school: 26.4%). They answered four questionnaires measuring their professional identity, work engagement, psychological capital, and burnout. PROCESS macro (SPSS 21.0) was used to conduct mediation analyses of work engagement and psychological capital in the relationship between professional identity and burnout. Working simultaneously, work engagement, and psychological capital partially mediated the aforementioned relationship, which could reduce burnout. Working sequentially completely mediated the relationship between professional identity and burnout, and hence, the latter was the lowest. Specific implications are discussed, such as the improvement of professional identity and psychological capital.

## 1. Introduction

### 1.1. Teacher Burnout

Teacher burnout, defined as physical and mental exhaustion caused by long-term work pressure, lack of coping resources, and ability to deal with stress in the teaching situation [1], is a worldwide problem [2,3]. Burnout negatively impacts teachers’ physical and psychological well-being, teaching quality, and relationships with children [4,5]. Compared with college teachers, those in primary and secondary schools are often under the pressure of long working hours, work–family conflict, lack of social support, and difficulties in classroom management. Thus, they are more vulnerable to stress and burnout [6]. The burnout level of female primary and secondary school teachers in China is high [7]. The research conducted by Wu et al. (2019) found that they scored high on the dimensions of emotional exhaustion and de-personalization in a sample of 5672 participants from 20 regions across the country. Thus, it is important to identify protective factors and explore the prevention mechanisms of burnout among female teachers.

### 1.2. Teacher Professional Identity and Burnout

Existing research has shown that professional identity is regarded as a protective factor for preventing burnout among teachers [8,9,10], police officers [11], and doctors [12]. Teachers’ professional identity is defined as an individual’s desire and love for the continuous work required by the profession [13]. Social identity theory posits that individuals’ identities stemming from group membership are essential for their self-concepts [14], which, in turn, impact their emotions, cognitions, and behavior [15,16]. Conservation of resource theory (1993) posits that an individual suffers from job burnout when the work requirements cannot be fully met and when the individual loses specific resources [17]. Teachers’ professional identity, as a psychological resource, could compensate for the loss of resources caused by work, thus reducing job burnout. Although teachers’ professional identity positively impacts the prevention of burnout, little is known about the mediating mechanism underlying their relationship. Thus, possible mediating mechanisms are discussed in our research.

### 1.3. Mediating Role of Work Engagement

Work engagement is defined as “a positive, fulfilling, work-related state of mind characterized by vigor, dedication, and absorption” [18,19], which might mediate the relationship between professional identity and burnout. Research has shown that professionalism, which is negatively related to burnout, is a significant predictor of work engagement, and the latter is negatively related to burnout [20,21]. Meanwhile, from the perspective of the psychological contract theory, teachers’ professional identity is a dimension of psychological capital (PsyCap) [22]. If teachers have an identity attached to their job, they are more likely to invest more in their career choices and, hence, are likely to experience more work engagement and less job burnout [23]. Based on theory and empirical evidence, this study hypothesized that the relationship between their professional identity and burnout is mediated by work engagement.

### 1.4. Mediating Role of PsyCap

Moreover, the relationship between teachers’ professional identity and burnout might be mediated by their PsyCap. It is defined as an individual’s positive psychological state of development [24]. The job demands–resources (JD-R) model posits that job characteristics can be divided into requirements and resources [25]. Job requirements are environmental stressors [26], such as workload, time pressure, and work–family conflict, while resources involve the physical, psychological, social, and organizational aspects of work, such as social support, rewards, and other external resources. The JD-R model posits that job resources can increase work motivation and result in low burnout. PsyCap can have a positive effect on lowering burnout [27,28]. Meanwhile, research has shown that professional identity positively affects PsyCap [29]. According to the theory and research hypothesis, this study hypothesized that the relationship between teachers’ professional identity and burnout could be mediated by positive psychological elements.

### 1.5. Sequential Mediating Roles of Work Engagement and PsyCap

Furthermore, work engagement and PsyCap could sequentially mediate the relationship between teachers’ professional identity and burnout. Research has shown that work engagement can be predicted by professional identity and is associated with PsyCap [30,31]. A longitudinal study found that PsyCap can be affected by work engagement [32]. According to the JD-R model, job resources can be divided into personal and work resources [25]. In this study, work engagement is a work resource and PsycCap is a personal resource. Work-related resources can facilitate the building of PsyCap to a certain extent [32]. PsyCap can trigger an individual’s intrinsic motivation, thereby reducing the burnout level [33]. Thus, this study hypothesizes that work engagement and PscyCap mediate the relationship between teachers’ professional identity and burnout sequentially.

### 1.6. Present Study

**Figure 1:**
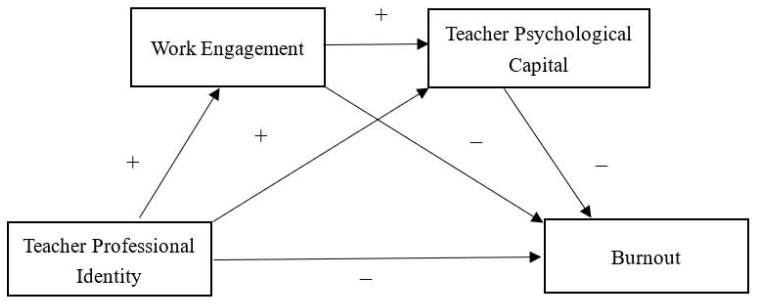
Hypothesized model.

Based on these theories and empirical evidence, this study aimed to test the mediating roles of work engagement and PsyCap in the relationship between teachers’ professional identity and burnout, simultaneously and sequentially. This study hypothesized that the relationship between professional identity and burnout is mediated by work engagement (H1), the relationship between professional identity and burnout could be mediated by positive PsyCap (H2), and work engagement and PsyCap mediated the relationship between professional identity and burnout sequentially (H3). The hypothesized model is illustrated in Figure 1.

## 2. Method and Materials

This study was approved by the ethics committee of the Zhejiang Normal University, and the approval number was ZSRT2022020. It was conducted according to the Declaration of Helsinki and APA ethical standards. Only female teachers from kindergarten and primary and secondary schools were chosen to participate. This was for two reasons. First, teachers in primary and secondary schools face the pressure of long working hours, work–family conflict, and difficulties in classroom management more frequently compared to college teachers. Second, the burnout level of female primary and secondary school teachers in China is high. Although equality between men and women is emphasized in the present society, it is undeniable that women play a bigger role in the family and take on more family responsibilities in China, which could lead to job burnout. Thus, we chose female primary and secondary school teachers as our participants.

The questionnaire link was sent to the participants using the Credamo platform, which was used to record responses and store data. The link was sent to teachers by their principals, who participated in a training program aimed at one district in Zhejiang Province. Simple sampling was used, and data were collected online. Informed consent was provided by the participants online. If they agreed to participate, they could choose an option and continue to fill out these questionnaires. If they did not want to participate, they could give up. Then, SPSS 21.0 was used for further analysis. Based on the principle of voluntary participation, this study did not provide any reward to the participants.

The original sample comprised 2465 female teachers from a city in Zhejiang Province. After the screening, 2220 valid responses (response rate: 90%) were included in the analysis. The final sample included 376 kindergarten teachers (16.9%), 1259 primary school teachers (56.7%), and 585 secondary school teachers (26.4%). The average age was 39 years (*SD* = 8.74). The participants had been teaching for an average of 17.90 years.

## 3. Measures

### 3.1. Teacher Professional Identity

This was measured using the teachers’ professional identity scale, which was designed by Wei, Song, and Zhang (2013) [34]. The scale includes four dimensions (i.e., occupational values, role values, sense of occupational belonging, and professional behavior inclination) and 18 items. The items were scored on a five-point Likert scale (1 = strongly disagree, 5 = strongly agree). The internal consistency coefficient of the scale was 0.94. The CFA results showed *χ*^2^/*df* = 3.98, CFI = 0.99, AGFI = 0.98, TLI = 0.99, and RMSEA = 0.03 (90% CI = 0.03, 0.04), indicating that this tool has good validity in our research.

### 3.2. Work Engagement

This was measured using the Utrecht work engagement scale (UWES-9) developed by Schaufeli and Bakker (2003) [35], which comprises three dimensions (vigor, dedication, and absorption). This scale includes nine items. The items were rated on a five-point Likert scale (1 = never, 5 = always). The internal consistency coefficient was 0.94. The CFA results showed *χ*^2^/*df* = 4.90, CFI = 0.99, AGFI = 0.98, TLI = 0.99, and RMSEA = 0.04 (90% CI = 0.02, 0.05), indicating that this tool has good validity in our research.

### 3.3. Teacher Psychological Capital

This was measured using the PsyCap scale developed by Luthans et al. (2004) [36], which comprises four dimensions (confidence, hope, optimism, and resilience). This scale included 19 items. All items are rated on a six-point Likert scale (1= strongly disagree, 6 = strongly agree). The internal consistency coefficient was 0.93. The CFA results showed *χ*^2^/*df* = 3.27, CFI = 0.99, AGFI = 0.98, TLI = 0.99, and RMSEA = 0.03 (90% CI = 0.02, 0.03), indicating that this tool has good validity in our research.

### 3.4. Burnout

This was measured using the professional quality of life scale designed by Stamm (2010) [37]. It includes three dimensions: compassion satisfaction, burnout, and secondary traumatic stress. The burnout subscale includes eight items rated on a five-point Likert scale (1 = never, 5 = very often), with higher scores indicating higher burnout levels. The Cronbach’s alpha was 0.90. The CFA result showed that *χ*^2^/*df* = 3.91, NFI = 0.99, AGFI = 0.99, TLI = 0.99, and RMSEA = 0.03 (90% CI = 0.01, 0.05), indicating the validity of this tool.

## 4. Data Analyses

SPSS 21.0 was used to test the mediating roles of work engagement and PsyCap in the relationship between professional identity and burnout among female teachers. Model 4 of the PROCESS macro in SPSS was used to test the single mediation of work engagement and PsyCap on the relationship between teachers’ professional identity and burnout. Model 6 of the PROCESS macro in SPSS was used to test the multiple mediation effects of work engagement and PsyCap on this relationship.

## 5. Results

### 5.1. Results of Common Method Bias

As the data were collected using a self-reported questionnaire, it was necessary to test the common methodological deviations. The Harmon single-factor test was used for this purpose. The results showed that the eigenvalues of the 10 factors were greater than one, and the explanatory power of the first factor was 38.98%, which was less than the critical value of 40%. This indicated that there were no serious problems resulting from the common method bias.

### 5.2. Correlational Analysis

Table 1 presents the results of the descriptive and correlational analyses. These results showed that burnout was negatively correlated with teachers’ professional identity (*r* = −0.55, *p* < 0.001), work engagement (*r* = −0.68, *p* < 0.001), and PsyCap (*r* = −0.76, *p* < 0.001), and teachers’ professional identity was positively related to work engagement (*r* = 0.63, *p* < 0.001) and PsyCap (*r* = 0.65, *p* < 0.001).

### 5.3. Mediation Analysis of Work Engagement

The mediation analysis of work engagement on the relationship between teachers’ professional identity and burnout is shown in Table 2. The results showed that professional identity had a positive significant effect on work engagement (*B* = 1.01, *p* < 0.001, 95% CI = [0.96, 1.06]), and the latter had a negative significant effect on burnout (*B* = −0.50, *p* < 0.001, 95% CI = [−0.53, −0.47]). Meanwhile, teachers’ professional identity also had a significant negative effect on burnout (*B* = −0.28, *p* < 0.001, 95% CI = [−0.34, −0.23]), suggesting that work engagement had a partial effect. Indirect effects accounted for 63.93% of the total effect.

### 5.4. Mediation Analysis of Teacher PsyCap

The mediation analysis of teachers’ PsyCap on the relationship between professional identity and burnout among teachers is shown in Table 3. It was found that professional identity had a significant positive effect on PsyCap (*B* = 1.02, *p* < 0.001, 95% CI = [0.97, 1.07]), and the latter had a significant negative effect on burnout (*B* = −0.64, *p* < 0.001, 95% CI = [−0.67, −0.60]). Meanwhile, professional identity also had a significant negative effect on burnout (*B* = −0.14, *p* < 0.001, 95% CI= [−0.19, −0.09]), suggesting that PsyCap had a partial effect on the relationship. Indirect effects accounted for 82.24% of the total effect.

### 5.5. Multiple Mediation Analyses of Work Engagement and PsyCap

The results of the multiple mediation analyses of work engagement and teacher PsyCap are shown in Table 4. The results showed that the pathways for “teachers’ professional identity → work engagement → burnout” (indirect effect = −0.16, 95% CI = −0.19 to −0.13) and “teachers’ professional identity → PsyCap → burnout” (indirect effect = −0.16, 95% CI = −0.19, −0.14) were significant, indicating that work engagement and PsyCap mediate the relationship between professional identity and burnout among female teachers in China. Similarly, the sequential pathway for “teachers’ professional identity → work engagement → PsyCap → burnout” was significant (indirect effect = −0.20, 95% CI = −0.22, −0.18), implying that a high level of professional identity was sequentially associated with higher work engagement (*B* = 1.01, *p* < 0.001), higher PsyCap (*B* = 0.55, *p* < 0.001), and lower burnout (*B* = −0.51, *p* < 0.001). However, the residual direct pathway for “teachers’ professional identity → burnout” was not significant (*B* = −0.05, *p* > 0.05). Thus, work engagement and PsyCap mediated the relationship between professional identity and burnout, simultaneously and sequentially. When they work sequentially, this relationship is completely mediated. Overall, this multiple mediation model accounted for a significant amount of variance in burnout among female primary and secondary school teachers in China (*R*^2^ = 0.21).

## 6. Discussion

Based on theoretical and empirical evidence, this study proposes a hypothesized model. The relationship between teachers’ professional identity and burnout might be mediated by work engagement and PsyCap, simultaneously and sequentially. These results support our hypotheses. The results showed that burnout was negatively predicted by teachers’ professional identity, work engagement, and PsyCap. These results indicate that, when work engagement and PsyCap operate separately, the relationship between professional identity and burnout is partially mediated. However, when work engagement and PsyCap work sequentially, the relationship between professional identity and burnout is completely mediated. These results are discussed below.

### 6.1. Single Mediation Effect of Work Engagement

This study found that work engagement mediated the relationship between teachers’ professional identity and burnout. This result is similar to that of some previous studies [35,38,39]. The research conducted by Salanova and Schaufeli (2008) found that work engagement mediates the relationship between job resources and proactive behavior. In this study, professional identity was regarded as an essential resource. A possible explanation for the mediating role of work engagement is that it is intrinsic motivation [39]. When an individual has a strong job identity, they have a strong intrinsic motivation to engage in their work, have good work performance, and reduce their burnout level.

### 6.2. Single Mediation Effect of PsyCap

This study found that teachers’ PsyCap mediates the relationship between professional identity and burnout, supporting H2. This result is similar to that of previous studies [40,41,42]. For instance, the research conducted by Mohagheghi et al. (2015) found that PsyCap can mediate the relationship between job demands and burnout. A possible explanation for its mediating role is that professional identity boosts their PsyCap, which can reduce burnout. PsyCap represents the combination of self-efficacy, resilience, optimism, and hope, which are four state-like positive psychological constructs [43]. The JD-R model posits that job resources can trigger work motivation, strengthen self-efficacy [44], and reduce burnout levels [25].

### 6.3. Sequential Mediation Effects of Work Engagement and PsyCap

An interesting result was that work engagement and teachers’ PsyCap mediated the relationship between professional identity and burnout when they operated sequentially. When work engagement and PsyCap operated separately, the direct effect of professional identity on burnout was also significant, indicating that work engagement and PsyCap played a partial mediating role in the relationship between professional identity and burnout when they worked separately. However, when they work sequentially, the pattern of the results is different. The direct effect of professional identity on burnout was not significant when they worked sequentially in their relationship, which indicates that the relationship between professional identity and burnout was completely mediated by work engagement and PsyCap. These results support our hypothesized model. This result is similar to those of previous studies [39,40]. An explanation may be that professional identity activates work engagement, and the latter has the benefit of building PsyCap [32]. A longitudinal study found that work engagement could predict subsequent PsyCap [32]. According to the JD-R model, job resources can be divided into personal and work resources [25]. In this study, work engagement is a work resource and PsyCap is a personal resource. Work-related resources can facilitate the building of PsyCap to a certain extent [32]. PsyCap can trigger an individual’s intrinsic motivation, thereby reducing the burnout level [33].

### 6.4. Theoretical and Practical Implications

The results have several theoretical and practical implications. Theoretically, this study tested the mediating mechanisms underlying the relationship between professional identity and burnout among female primary and secondary school teachers, enriching research on their burnout. Meanwhile, the results support the conservation of resources theory and the JD-R model. This study indicated that emphasizing professional identity could reduce burnout. Specifically, two approaches can be adopted. First, the department of education and school administrators should focus on the role of training in improving professional identity. In the training of pre-service teachers and the annual training of in-service teachers, along with the improvement of their professional skills, teachers’ professional identity must be included in the training theme. Second, the importance of the cultural atmosphere in improving teachers’ professional identity should also be emphasized. The tradition of respecting and loving teachers should be promoted in society, which has great benefits in strengthening professional identity. However, increasing teachers’ work engagement and strengthening PsyCap may be effective in preventing burnout. Performance bonuses can play a vital role in improving teachers’ work engagement. Many teachers felt that there was an imbalance between their investment and return, with the former significantly outweighing the latter. Therefore, a modest increase in pay may improve teachers’ job engagement. Moreover, organizational support from schools could help teachers invest more effort in their work because it can buffer the stress from family, work, and society. Interventions can be used to improve teachers’ PsyCap. A PsyCap intervention model designed by Luthans has been shown to significantly improve it, which can be generalized by education departments and implemented by school managers. In addition, PsyCap mainly includes four dimensions: self-efficacy, hope, optimism, and resilience. Thus, other interventions could be designed to improve these four dimensions regarding teachers. Preventing or reducing the burnout level among teachers is a complex issue that requires effort from society.

### 6.5. Limitations and Future Directions

This study has some limitations and requires further research. First, it used a cross-sectional design to test these relationships, resulting in a deficiency in exploring causal relationships. Future research could use a longitudinal design to test these relationships. Second, this study only tested the mediating factors but did not explore the moderating factors. Some factors that can prevent burnout among female primary and secondary school teachers should be considered in future research. Third, this study only explored female teachers’ professional identity, work engagement, PsyCap, and burnout, and hence, it is not possible to compare the differences between male and female teachers. Although the manipulation resulted in a lack of interesting results, it provided directions for future research. Future research could explore the differences between male and female teachers to provide relevant coping strategies for both.

## 7. Conclusions

This study tested the mechanism underlying the relationship between professional identity and burnout among female teachers from kindergartens and primary, and secondary schools in China based on theories and empirical evidence. This study found that this relationship can be mediated by work engagement and PsyCap, simultaneously and sequentially.

When work engagement and PsyCap worked separately, the relationship between teachers’ professional identity and burnout was partially mediated. However, when work engagement and PsyCap worked sequentially, this relationship was completely mediated by the two factors. These results have implications for preventing burnout and reducing its level among primary and secondary school teachers.

## Figures and Tables

**Table 1:** Means standard deviations, and bivariate correlations among variables.

Variables	*M*	*SD*	1	2	3	4
TPI	4.51	0.48	--			
WE	3.84	0.77	0.63 ***	--		
TPC	4.76	0.76	0.65 ***	0.75 ***	--	
BO	2.17	0.69	−0.55 ***	−0.68 ***	−0.76 ***	--

Note. TPI = teacher professional identity, WE = work engagement, TPC = teacher psychological capital, BO = burnout. *** *p* < 0.001.

**Table 2:** Results of mediation analysis of work engagement.

	M: WE			Y: BO		
	*B*	*SE*	95% CI	*B*	*SE*	95% CI
X: TPI	1.01 ***	0.03	0.96, 1.06	−0.28 ***	0.03	−0.34, −0.23
M: WE	--	--	--	−0.50 ***	0.02	−0.53, −0.47
Constant	−0.71 ***	0.12	−0.94, −0.47	5.37 ***	0.10	5.17, 5.56
*R*^2^ = 0.40				*R*^2^ = 0.49		
*F* (12,218) = 1452.85 ***				*F* (22,217) = 1073.73 ***		

Note. TPI = teacher professional identity, WE = work engagement, BO = burnout. *** *p* < 0.001.

**Table 3:** Results of mediation analysis of teacher psychological capital.

	M: TPC			Y: BO		
	*B*	*SE*	95% CI	*B*	*SE*	95% CI
X: TPI	1.02 ***	0.03	0.97, 1.07	−0.14 ***	0.03	−0.19, −0.09
M: TPC	--	--	--	−0.64 ***	0.02	−0.67, −0.60
Constant	0.16 ***	0.12	−0.07, 0.38	5.82 ***	0.09	5.65, 6.00
*R*^2^ = 0.42				*R*^2^ = 0.59		
*F* (12,218) = 1592.60***				*F* (22,217) = 1585.12 ***		

Note. TPI = teacher professional identity, TPC = teacher psychological capital, BO = burnout. *** *p* < 0.001.

**Table 4:** Testing the pathways of the multiple mediation model.

Effect	*B*	*SE*	95% CI	
**Direct effects**				
TPI → WE	1.01 ***	0.03	0.96	1.06
TPI → TPC	0.46 ***	0.03	0.41	0.52
WE → TPC	0.55 ***	0.02	0.52	0.58
TPI → BO	−0.05	0.03	−0.10	0.00
WE → BO	−0.22 ***	0.02	−0.26	−0.19
TPC→BO	−0.51 ***	0.02	−0.54	−0.47
**Indirect effects**				
TPI → WE → BO	−0.16	0.02	−0.19	−0.13
TPI → TPC → BO	−0.16	0.01	−0.19	−0.14
TPI → WE → TPC → BO	−0.20	0.01	−0.22	−0.18

Note. TPI = teacher professional identity, WE = work engagement, TPC = teacher psychological capital, BO = burnout. *** *p* < 0.001. → means “predicts”.

## Data Availability

Data can be obtained with the consent of the corresponding author.
